# The Relationship Between Health Changes and Community Health Screening Participation Among Older People

**DOI:** 10.3389/fpubh.2022.870157

**Published:** 2022-04-27

**Authors:** Benfeng Du, Yuexuan Mu

**Affiliations:** ^1^Interdisciplinary Innovation Platform of Public Health and Disease Prevention and Control for Health Policy, Renmin University of China, Beijing, China; ^2^School of Sociology and Population Studies, Renmin University of China, Beijing, China

**Keywords:** older people, community health screening, health promotion, fixed effects model, health change

## Abstract

The utilization of health screening and other community health services has been a hot topic in China. Thus, this study examined the effect of health changes (self-rated health, physical health, and mental health) on older people's community health screening participation in China. We derived the data from the 2016 and 2018 waves of the Chinese Longitudinal Aging Social Survey (CLASS). This paper included 10,992 observations in two waves. We tested the causal relationship using the fixed effects model. Approximately 29.56% of the respondents participated in a health screening. Notably, after controlling for covariates, changes in mental and physical health both significantly influenced seniors' participation in health screenings (self-rated health: β = 0.188, 95% CI [−0.037, −0.413]; physical health: β = 0.078, 95% CI [0.032, −0.124]; mental health: β = 0.034, 95% CI [−0.057, −0.002]). The findings showed age, educational level, income level, and family support to be significant factors associated with community health screening participation. Additionally, we identified a partial mediating effect of mental health between self-rated health and health screening participation and a partial mediating effect of depression between physical health and health screening participation. The results highlight the important role of health changes in influencing participation and promoting health screening in China. On this basis, healthcare providers in the community may consider health changes as a screening criterion to promote health screening, guiding other health promotion and prevention programs while promoting healthy aging.

## Introduction

Successful aging originates from healthy aging. Healthy aging requires older people to improve and maintain their health (1). Poor health conditions increase healthcare costs and the caregiving burden (2). Primary health services for older adults are beneficial to improving the quality of life and preventing disease. However, it is difficult for community healthcare centers to target different reasons behind an uninvolved population, improve the participation initiative of older people, and expand the health service coverage.

Health screening is a primary preventive service in the community. It helps identify potential risk factors older people face, improve the effective use of health services, and carry out daily health management (3). The Chinese health department requires each community to conduct free health screening once a year for people over 65 years. In practice, most communities have made the policy more flexible to cover residents over 60. Previous studies have shown a negative association between health screening utilization and healthcare expenditures among older people (4). Thus, it is a valuable academic topic to increase the participation rate in health screening (5). Recent studies focus on reviews or reports. Empirical studies always pay attention to the health or economic effects of health screening utilization (6). However, few studies have investigated the role of health in promoting the participation of health screening services among community residents.

According to the Andersen healthcare utilization model, four dimensions influence healthcare utilization: environment, demographic characteristics, health behaviors, and health outcomes (7). In addition, the sixth iteration of the Andersen model considers the individual as the analytical unit. It goes beyond healthcare utilization to include health outcomes as the endpoint of interest. This model illustrates how health outcomes may influence health beliefs and needs with feedback loops. The Anderson model can provide theoretical support for using community health screening services.

Based on the Andersen model, utilization of health services affects health outcomes. Some studies argued older people with poor physical conditions would be unwilling to use healthcare services (8). However, other studies considered that healthy people have weak prevention awareness, so they are less likely to participate in health programs (9). Research also suggests that older adults inevitably experience discomfort due to the loss of social roles and support. From a psychological perspective, older people experience depression and anxiety after retirement from social production. This adverse mental status hinders their participation in community programs (10); thus, physical and mental health may interact with each other. For instance, limited physical ability may lead to negative self-rated health (11). The poor physical condition of older people may also lead to some psychological problems that prevent them from participating in community health programs (12). Therefore, we can infer the need for exploring internal mechanisms between different aspects of health factors.

Previous studies have mainly used health-related variables as dependent variables or applied only one dimension of health to examine its impact on health service utilization. As a result, these studies lack a multidimensional examination of health influence on health service participation. In this study, we divided health changes into three dimensions: self-rated health (SRH), physical health, and mental health, to examine the effects of health changes in each dimension on changes in community health screening participation. First, we analyzed the impact of health changes on health screening participation based on different health dimensions. Second, if we found an influence, we further analyzed whether there is an influence mechanism between different health dimensions and health screening participation, such as a mediation or moderation effect. Finally, we provided suggestions to improve the utilization of community health services.

This study's contributions are as follows. First, where previous studies focused on health screening focus in the clinical area, this study describes the current participation rate of health screening and improved participation rate from a public health perspective. Second, this study used panel data to explore the impact of health changes on participation changes from a dynamic perspective. Third, this study also argues for an internal mechanism between the three health dimensions, which can help community workers pinpoint the health status of non-participants more rigorously from a practical perspective.

## Method

### Sampling

We selected data from the 2016 and 2018 Chinese Longitudinal Aging Social Survey (CLASS). Class is a national and continuous large-scale social survey project designed by Renmin University of China's Institute of Gerontology. The baseline survey included 11,511 respondents in 2014. The first follow-up survey traced 6,583 older people from the baseline survey and added 4,888 new samples in 2016. The second follow-up survey traced 9,672 samples, and 1,746 samples were new in 2018. The 2016 survey introduced an investigation of community health services, so we only used data from the 2016 and 2018 surveys. After removing missing samples, newly added samples in 2018 (to avoid non-uniform weights for different time point groups), and missing data in main variables, we had 10,992 observations finally.

### Measurements

#### Dependent Variable

The dependent variable was participation in community health screening. We measured it by the experience of free community health screening participation in the past 12 months (1 = *yes*; 0 = *no*).

#### Independent Variables

We measured health changes with three variables: physical health, mental health, and self-rated health (SRH). We used the score of activities of daily living (ADL) to measure physical health. Furthermore, the CLASS questionnaire uses the Lawton scale to evaluate ADL (13). This scale has 14 items to identify respondents' ability to bathe, dress, go to the toilet, eat, visit a neighbor's house, wash clothes, and cook. Each item has three selections (1 = *No need for help from others*, 2 = *Some help is needed*, 3 = *I can't do it at all*). We added 14 items and obtained the total score of ADL. The higher the score, the poorer the individual's ability to perform the activities of daily living. The Cronbach's α coefficient of the scale was 0.912.

To measure mental health, we used the “Center for Epidemiological Studies Depression Scale” (14) to report negative psychological experiences in the last week. The scale includes nine items. The higher the total score, the more severe the depression. The Cronbach's α coefficient of the scale was 0.756.

We asked, “How do you feel about your health compared with your peers?” to measure SRH. The question response range was 1 = *Very good*, 2 = *good*, 3 = *fair*, 4 = *not good*, 5 = *very bad*. We merged the first two categories to represent “healthy” and the latter three categories to represent “unhealthy.”

#### Covariates

Covariates included demographic factors and social factors. Demographic factors were age, gender, residence (0 = *rural*, 1 = *urban*), marital status (0 = *unmarried*, 1 = *married*), education level (0 = *illiterate*, 1 = *primary school*, 2 = *middle and high school*, 3 = *junior college and above*), income (the logarithm of annual income). The social factors included living with children (0 = *No*, 1 = *Yes*), family support, friend support, and community type (1 = *neighborhood community*, 2 = *mixed unit community*, 3 = *affordable housing communities*, 4 = *commercial housing complex*, 5 = *high-end residential area*, 6 = *urban village*, 7 = *rural*).

### Data Analysis

#### Model Selection

Mixed regression, random effects, and fixed effects models are all different strategies with longitudinal data. Mixed regression only aggregates all time points and assumes no individual effects. We drop mixed regression model because it only expands the sample size and ignores the omitted heterogeneity among individuals. The fixed effects and random effects models can solve the estimation bias of missing variables. The main difference is that the fixed-effects model requires the explanatory variables to vary over time, while the random-effects model requires as many control variables as possible that do not vary over time. In this study, the key independent variables all vary over time, but there are also many control variables that do not vary over time. Thus, we further performed the Hausman test to determine the final model and found that the fixed effects model was more suitable than the random effects model (*p* < 0.001).

The specific model settings are as follows:


(1) $$\begin{array}{l} \ln\left(\frac{p_{i1}}{1-p_{i1}}\right)=u_{i1}+\beta_{1}health_{i1}+\beta_{2}controlA_{i1}\\ \;+\beta_{3}controlB_{i}+\nu_{i}+\varepsilon_{i1} \end{array}$$

(2) $$\begin{array}{l} \ln\left(\frac{p_{i2}}{1-p_{i2}}\right)=u_{i2}+\beta_{2}health_{i2}+\beta_{2}controlA_{i2}\\ \;+\beta_{3}controlB_{i}+\nu_{i}+\varepsilon_{i2} \end{array}$$

We use a subtraction formula: Equation (2)-Equation (1):


(3) $$\begin{array}{l} \ln\left[\mathrm{Pr}(y_{i1}=0,y_{i2}=1)/\mathrm{Pr}(y_{i1}=1,y_{i2}=0)\right]=\left(u_{i2}-u_{i1}\right)\\ +\beta_{1}\left(health{\;}_{i2}-health{\;}_{i1}\right)+\beta_{2}\left(\;control\;A_{i2}-\;control\;A_{i1}\right)\\ \;+(\varepsilon_{i2}-\varepsilon_{i1}) \end{array}$$

Equation (1) represents the model from the 2016 wave in the survey (t = 1), whilst equation (2) represents the model from the 2018 wave (t = 2). *y*_*it*_ represents whether individual *i*will participate in the community health screening during the two periods, *y* has only two values, 0 and 1. *p*_*it*_ is the probability when *y*_*it*_=1. *u*_*it*_ represents the intercept over time. *health*_*it*_ represents the independent variables at two time periods, *controlA*_*i*_ represents the control variable that varies with time, including age, marital status, economic level, family support and friend support, *controlB*_*i*_represents the control variable that does not change over time, including gender, education level, community type, cohabitation, and place of residence. ν_*i*_ represents the unobserved heterogeneity, which is regarded as a fixed parameter that does not change with time. ε_*it*_ represents the random error that changes over time. β_1_β_2_β_3_ represents the influence of the explanatory variable on the dependent variable. It can be seen from equation (3) that the parts of the control variables and missing variables that do not change with time are all differentiated. We have achieved the main purpose of controlling other missing variables that have not been observed.

#### Analysis Process

First, we established three nested models to examine the impact of three dimensions of health change on the community health screening participation changes during the two time periods (2016 and 2018). Second, we performed mediation analysis to identify the internal relationship between three health variables in the health screening participation. Third, we used Stata and Mplus software for data analysis.

## Results

### Descriptive Analysis

Table 1 shows the basic information of variables. We found that community health screening increased from 25.56 to 33.55% in 2 years. In the total sample, the participation rate in health screening was 29.56%. The average score of ADL was 15.11, which represents a healthy physical condition of the older persons (the range of the ADL score was 14–40). However, the psychological status of the older people is not good, as the average score of depression was 14.44, and the range of depression scores was 0–27. In addition, while only 27.51% of the seniors ranked themselves as healthy, this percentage declined by 1.93% between 2016 and 2018.

**Table 1:** Basic information of all variables.

	**Total sample**		**2016**		**2018**	
	***N* (%)**	**Mean (SD)**	***N* (%)**	**Mean (SD)**	***N* (%)**	**Mean (SD)**
Participation in health screening	3,249 (29.56)	0.29 (0.46)	1,405 (25.56)	0.25 (0.44)	1,844 (33.55)	0.33 (0.47)
ADL		15.11 (2.88)		15.16 (2.81)		15.07 (2.96)
Depression		14.44 (3.50)		14.75 (3.70)		14.10 (3.24)
SRH (healthy)	3,024 (27.51)	0.27 (0.45)	1,565 (28.48)	0.28 (0.45)	1,459 (26.55)	0.26 (0.44)
Age		70.63 (7.27)		69.63 (7.20)		71.63 (7.20)
Gender (male)	5,504 (50.07)	1.50 (0.50)	2,752 (50.07)	1.50 (0.50)	2,752 (50.07)	1.50 (0.500)
Residence (urban)	7,059 (64.37)	0.64 (0.48)	3,546 (64.81)	0.65 (0.48)	3,513 (63.92)	0.64 (0.48)
Marital status (married)	7,910 (71.96)	0.72 (0.45)	4,041 (73.54)	0.73 (0.44)	3,868 (70.38)	0.70 (0.46)
Educ (illiterate)	3,640 (33.11)	2.06 (0.89)	2,125 (38.66)	2.01 (0.94)	1,515 (27.57)	2.12 (0.83)
Primary school	3,385 (30.80)		1,443 (26.26)		1,942 (35.33)	
High and middle school	3,573 (32.51)		1,653 (30.08)		1,920 (34.93)	
Junior college and above	394 (3.58)		275 (5.00)		119 (2.17)	
Income		8.80 (1.47)		9.24 (1.42)		8.38 (1.39)
Cohabit with children (Yes)	7,006 (63.74)	0.64 (0.48)		1.00 (0.00)		0.27 (0.45)
Family support		7.93 (3.52)		8.57 (4.06)		7.28 (2.72)
Friend support		6.89 (3.95)		7.47 (4.56)		6.31 (3.12)
Community type (neighborhood community)	2,099 (19.99)	4.49 (2.38)	1,030 (20.59)	4.43 (2.43)	1,069 (19.45)	4.54 (2.33)
Mixed unit community	993 (9.46)		586 (11.71)		407 (7.41)	
Affordable housing communities	162 (1.54)		92 (1.84)		70 (1.27)	
Commercial housing complex	2,375 (22.62)		968 (19.35)		1,407 (25.60)	
High-end residential area	106 (1.01)		61 (1.22)		45 (0.82)	
Urban village	846 (8.06)		368 (7.36)		478 (8.70)	
Rural	3,918 (37.32)		1,898 (37.94)		2,020 (36.75)	

The average age of the older people was 70.63. Both sexes were about equally represented in the analysis. Regarding education, 36.09% of the seniors reported at least a middle school education. Most seniors lived in the urban area (64.37%), remained married (71.96%), and lived with their children (63.74%). In Table 1, we reported the Logarithmic form of income; the average income of the older people was ¥15,473.51 per year. Based on State Statistics Bureau data, the national per capita income is ¥32,189 (15), so the economic condition of the older persons was a little lower than the overall level.

### Results From the Fixed Effects Model

Model 1 (shown in Table 2) examined the influence of health changes in SRH on survey participants' involvement in health screenings. The results showed that improvement of the SRH has a very significant positive impact on seniors' participation in health screenings (*sig*0.05). Compared with 2016, older people with good SRH in 2018 were more likely to participate in community health screening.

**Table 2:** The effect of health changes on community health screening participation.

**Variables**	**Model 1**			**Model 2**			**Model 3**		
	**Coef. (SE)**	***P*-Value**	**95% CI**	**Coef. (SE)**	***P*-Value**	**95% CI**	**Coef. (SE)**	***P*-Value**	**95% CI**
SRH	0.206 (0.104)	0.047	(0.003, 0.409)	0.130 (0.113)	0.249	(−0.091, 0.351)	0.188 (0.115)	0.102	(−0.037, 0.413)
Depression				−0.028 (0.014)	0.044	(−0.055, −0.001)	−0.029 (0.014)	0.034	(−0.057, −0.002)
ADL							0.078 (0.023)	0.001	(0.032, 0.124)
Age	0.643 (0.066)	0.000	(0.513, 0.774)	0.707 (0.072)	0.000	(0.565, 0.849)	0.722 (0.073)	0.000	(0.578, 0.865)
Residence	−0.546 (0.295)	0.064	(−1.126, 0.033)	−0.427 (0.322)	0.185	(−1.058, 0.204)	−0.432 (0.319)	0.176	(−1.058, 0.194)
Marital status (married)	0.360 (0.253)	0.154	(−0.135, 0.856)	0.381 (0.272)	0.162	(−0.153, 0.914)	0.474 (0.277)	0.087	(−0.069, 1.016)
Educ (illiterate)	−0.179 (0.072)	0.013	(−0.320, −0.038)	−0.213 (0.077)	0.006	(−0.364, −0.061)	−0.208 (0.078)	0.008	(−0.361, −0.055)
Income	0.075 (0.045)	0.097	(−0.014, 0.163)	0.108 (0.051)	0.035	(0.008, 0.208)	0.118 (0.052)	0.022	(0.017, 0.220)
Cohabit with children (Yes)	0.333 (0.147)	0.023	(0.045, 0.620)	0.281 (0.158)	0.075	(−0.029, 0.591)	0.290 (0.159)	0.068	(−0.021, 0.601)
Family support	0.087 (0.016)	0.000	(0.055, 0.119)	0.082 (0.018)	0.000	(0.047, 0.117)	0.080 (0.018)	0.000	(0.045, 0.115)
Friend support	−0.013 (0.015)	0.385	(−0.042, 0.016)	0.006 (0.016)	0.712	(−0.026, 0.038)	0.006 (0.016)	0.700	(−0.026, 0.038)
Community type (neighborhood community)	−0.030 (0.037)	0.415	(−0.102, 0.042)	−0.020 (0.038)	0.604	(−0.093, 0.058)	−0.015 (0.038)	0.695	(−0.091, 0.060)
LR Chi2	229.75			249.11			261.22		

^*^The gender variable did not differ between groups and was therefore automatically excluded by the model.

Model 2 introduced the depression score based on Model 1. The influence of SRH changes on dependent variables was no longer significant, and its coefficient was significantly reduced (from 0.206 to 0.130). Notably, the effect of change in mental health status on health screening participation was significant (*sig* < 0.05). Compared with 2016, older people with improved mental health in 2018 were more likely to participate in community health screening.

Model 3 included an ADL score based on Model 2. We found that the impact of SRH changes on the dependent variable was no longer significant, although the effects of mental health changes remained statistically significant (*sig* < 0.05). After including all health variables, the incidence ratio of older adults' participation in community health screening increased by 8% (*e*^0.078^−1 = 0.08), accompanied by a statistically significant one point increase in their ADL scores (*sig* < 0.05). This finding suggested that a decline in older adults' physical health can influence their decision to participate in community health screening.

In summary, the improvement across all three dimensions of health positively impacted the community health screening participation among older people. However, with the control of change in mental and physical health, the SRH coefficient changed and was no longer significant. In terms of other control variables, compared with 2016, older adults who enjoyed better living standards and better family relationships had an increased probability of participating in community health screening in 2018.

### Results From the Mediation Effect Model

**Figure 1:**
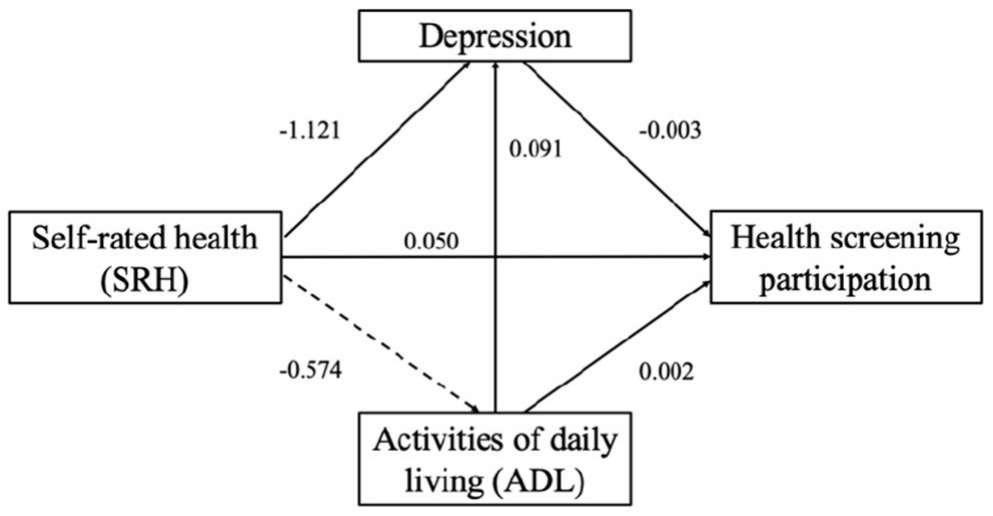
Structural equation model for the relationship between SRH, ADL, depression and health screening participations.

We found that health changes (SRH, ADL, and depression) affect health screening participation differently (see Table 2). SRH was significantly associated with health screening participation in Model 1 (Table 2), while the coefficient of SRH was insignificant after including ADL and depression. We performed a structural equation model (SEM) to test the internal relationship between three heath variables (16–19). Figure 1 shows a simplified version of the mediated model.

We tested the mediating effects with a bias-corrected bootstrap procedure (16, 17). If the 95% confidence interval for the indirect effect did not include 0, the mediating effect was statistically significant. On the other hand, if the 95% confidence interval for the direct effect included 0, it was fully mediated (Table 3).

**Table 3:** Bootstrapping test results for mediation effects.

**Path**	**95% CI**
SRH → depression → health screening participation		
Direct effect	0.023	0.065
Indirect effect	0.0001	0.006
SRH → ADL → health screening participation		
Direct effect	0.028	0.068
Indirect effect	−0.001	0.003
Depression → ADL → health screening participation		
Direct effect	−0.006	−0.001
Indirect effect	−0.0003	0.0001
ADL → depression → health screening participation		
Direct effect	−0.006	−0.001
Indirect effect	−0.001	−0.0001

Overall, we found a partial mediating effect of depression in SRH and health screening participation and no mediating effect of ADL in SRH and health screening participation. However, there was a partially mediated role of depression in ADL and health screening participation.

## Discussion

While life expectancy in China is increasing, older persons may spend more of their advanced years in poor health and living with disabilities. As a result, the demand for health services amongst older people is higher than in other groups. This demand is diverse, complex, and specialized. To transfer demand to effective utilization, many scholars have conducted studies on the current utilization of and participation in health services by older persons. However, against the backdrop of population aging in China, the mismatch between health service demand and health service utilization has led to health service non-utilization among older adults in China. With this in mind, this paper investigated the utilization of community health screening by older persons from the different health dimensions based on a nationally representative survey.

### Theoretical Implications

Consistent with the results from previous studies (20–22), the present study identified a significant relationship between age, education level, income, family support, and participation in health screening. Notably, socioeconomic factors were significant predictors of involvement in community health screening amongst older people. Age influences participation in community health screening, with relatively high participation rates among older seniors, a finding that is consistent with previous studies (23). However, the participation rate decreased the higher the individual's education level. We speculated that seniors with higher education levels are more health-conscious. Therefore, they demand higher quality and quantity of health screenings, and the community health screenings provide only basic screening items. We also found income level as another important influencing factor for health service utilization. However, although community health screenings are free for residents, our results still showed that the participation rate of low-income groups in community health screening was low. We propose two reasons for this: first, low-income groups do not know about the health screening program; second, the awareness of self-health management among low-income groups is insufficient.

In addition, this study provides new empirical evidence. We found that older adults' higher ADL scores and improvements in mental health and self-rated health can increase the participation rate in community health screening. However, we note that the relative roles of physical and mental health changes were greater. Meanwhile, there was a mediating effect of depression between SRH and community health screening participation. The results of the mediating effect indicate the importance of psychological adjustment for older adults, especially those with poor self-rated health. Psychology research has found that positive experiences and optimism can balance the frustration caused by negative events (e.g., physical illness). In addition, adopting effective multiple coping strategies can encourage the active participation of older adults in community activities.

### Limitations and Future Directions

Previous studies have generally only used cross-sectional data for analysis, lacking causal inference for the changes in different periods. In addition, previous research tends to choose one-dimensional indicators for health measurements, thus lacking comparisons of the extent and mechanisms of health effects on health screening participation in different dimensions—our research addressed this gap. However, this paper also has several limitations. First, the community health screening was free of charge, so its impact may differ from self-paying health screening when examining the impact of health changes on health screening participation. Due to the limits imposed by the questionnaire setting, future studies could compare two forms of health screening programs to understand factors influencing participation and adopt different strategies to promote health management among older people. Second, although this study applied panel data to analyze the causal relationship, the influencing factors were pre-set by the questionnaire. We suggest that future studies could explore a deeper range of influencing factors through in-depth interviews. Third, we lack the inclusion of other community health services variables, which may lead to some missing findings and limit the generalizability of our results. For instance, if an individual has other health issues and is seeing health professional(s) regularly, they are likely not to present for this type of generalist health screening. Further studies could include other health services as control variables.

### Practical Implications

This study has several important policy and intervention implications. First, the government should implement more psychological interventions for older adults with poor self-rated health to help them adopt positive and effective coping strategies, increasing their motivation to participate in community health programs. Second, we need to change the awareness of “remedial” participation. Studies have shown that older adults with deteriorating physical health will increase their engagement in community health screening. However, the community should disseminate preventive knowledge and transfer “remedial” to “preventive” participation by targeting those who have not developed problems or are in good health. Third, we recommend targeting low-income groups when designing community health screening plans because community health screening is free and should not be related to income level. However, the study results still show a positive association between income level and community health screening. Therefore, at the level of policy guidance, it is necessary to promote the keyword “free” and bolster the awareness of health management among low-income groups. In addition, GPs and their assistants can further promote this program when making follow-up visits.

## Conclusion

This study investigated the impact of health changes among older people on participation in community health screening. The findings suggest that mental health, physical health, age, educational level, income level, and family support promote older persons' participation in community health screening programs in China. Community health screening is a welfare policy, and it is free of charge. Participating in community health screening helps older persons understand their basic health conditions and lets their GPs know more about their needs to develop updated and precise prevention plans. Thus, the ultimate goal of the community is to have all seniors participate in the health screening program. This study can help communities target those groups that do not participate in the programs and recommend measures to promote their uptake.

## Data Availability Statement

The original contributions presented in the study are included in the article/supplementary materials, further inquiries can be directed to the corresponding author/s.

## Ethics Statement

The studies involving human participants were reviewed and approved by Renmin University of China. The patients/participants provided their written informed consent to participate in this study.

## Author Contributions

BD has undertaken data curation and methodology. YM has performed the formal analysis and finished the final editing. Both authors read and approved the final manuscript.

## Funding

This work was supported by the National Social Science Foundation Key Projects (grant number 21AZD073) and National Natural Science Foundation of China (grant number 71974194).

## Conflict of Interest

The authors declare that the research was conducted in the absence of any commercial or financial relationships that could be construed as a potential conflict of interest.

## Publisher's Note

All claims expressed in this article are solely those of the authors and do not necessarily represent those of their affiliated organizations, or those of the publisher, the editors and the reviewers. Any product that may be evaluated in this article, or claim that may be made by its manufacturer, is not guaranteed or endorsed by the publisher.
